# Supplementary material for: Single-Cell RNA Sequencing Reveals Molecular Features of Heterogeneity in the Murine Retinal Pigment Epithelium
Source: Int J Mol Sci. 2022 Sep 8;23(18):10419. doi: 10.3390/ijms231810419 (PMC9499471; doi:10.3390/ijms231810419)
Supplement: Supplementary file 1 [file ijms-23-10419-s001.zip › Figure S1.pdf]

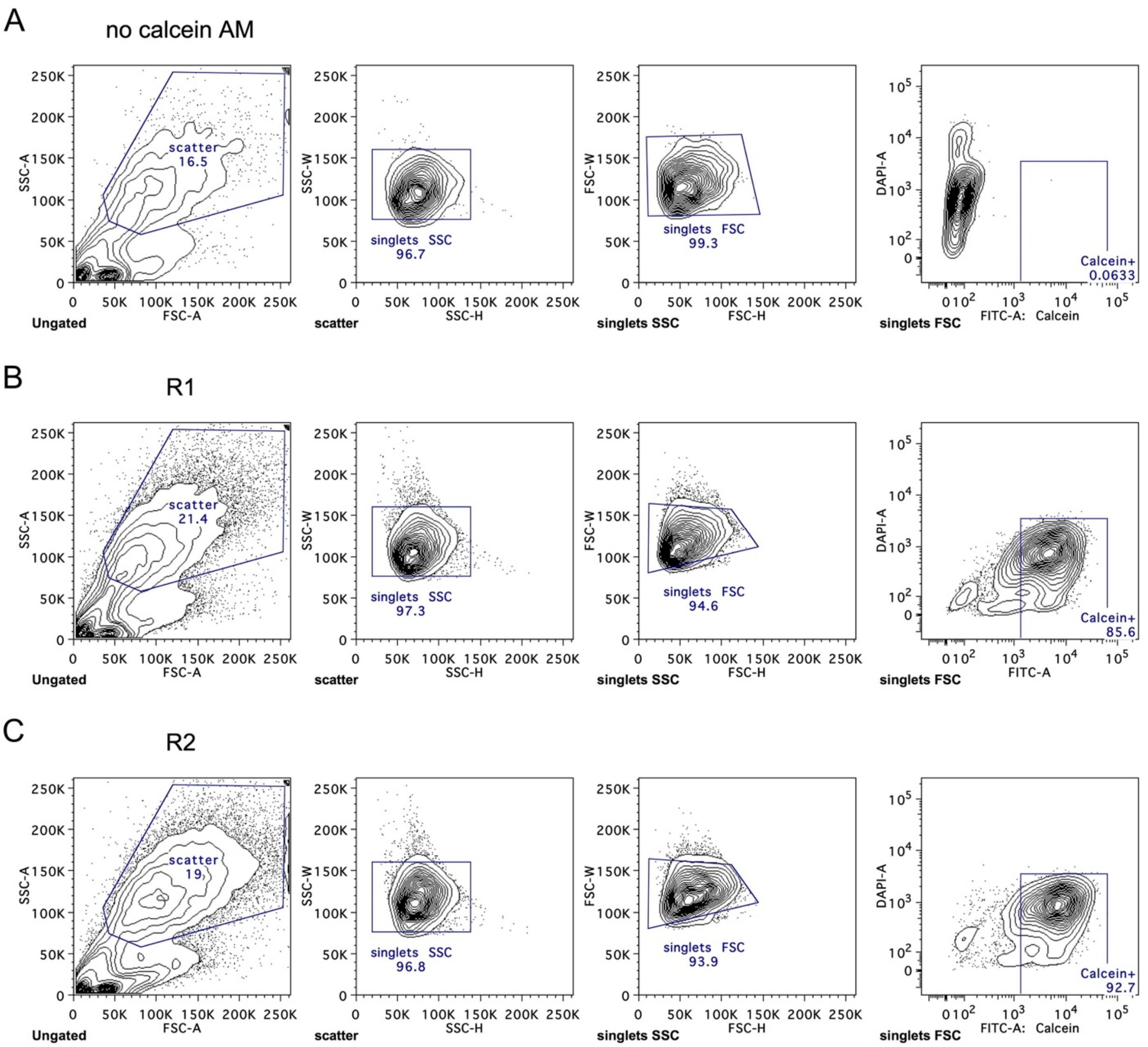

Figure S1. Fluorescence-activated cell sorting (FACS) gating strategy and yield. RPE cells isolated from mouse eyecups by enzymatic and mechanical disaggregation and stained with calcein-AM and DAPI were isolated by gating based on side scatter (SSC-A), forward scatter (FSC-A), and by gating for viable DAPI-negative and calcein-positive nucleated cells. (**A**) FACS of control samples without calcein-AM treatment showed few cells in the calcein-positive window. (**B**, **C**) FACS of the two replicates with added calcein-AM recovered a high percentage of viable cells from the SSA-A and FSC-A gated population.
