# Supplementary material for: Single-Cell RNA Sequencing Reveals Molecular Features of Heterogeneity in the Murine Retinal Pigment Epithelium
Source: Int J Mol Sci. 2022 Sep 8;23(18):10419. doi: 10.3390/ijms231810419 (PMC9499471; doi:10.3390/ijms231810419)
Supplement: Supplementary file 1 [file ijms-23-10419-s001.zip › Figure S2.pdf]

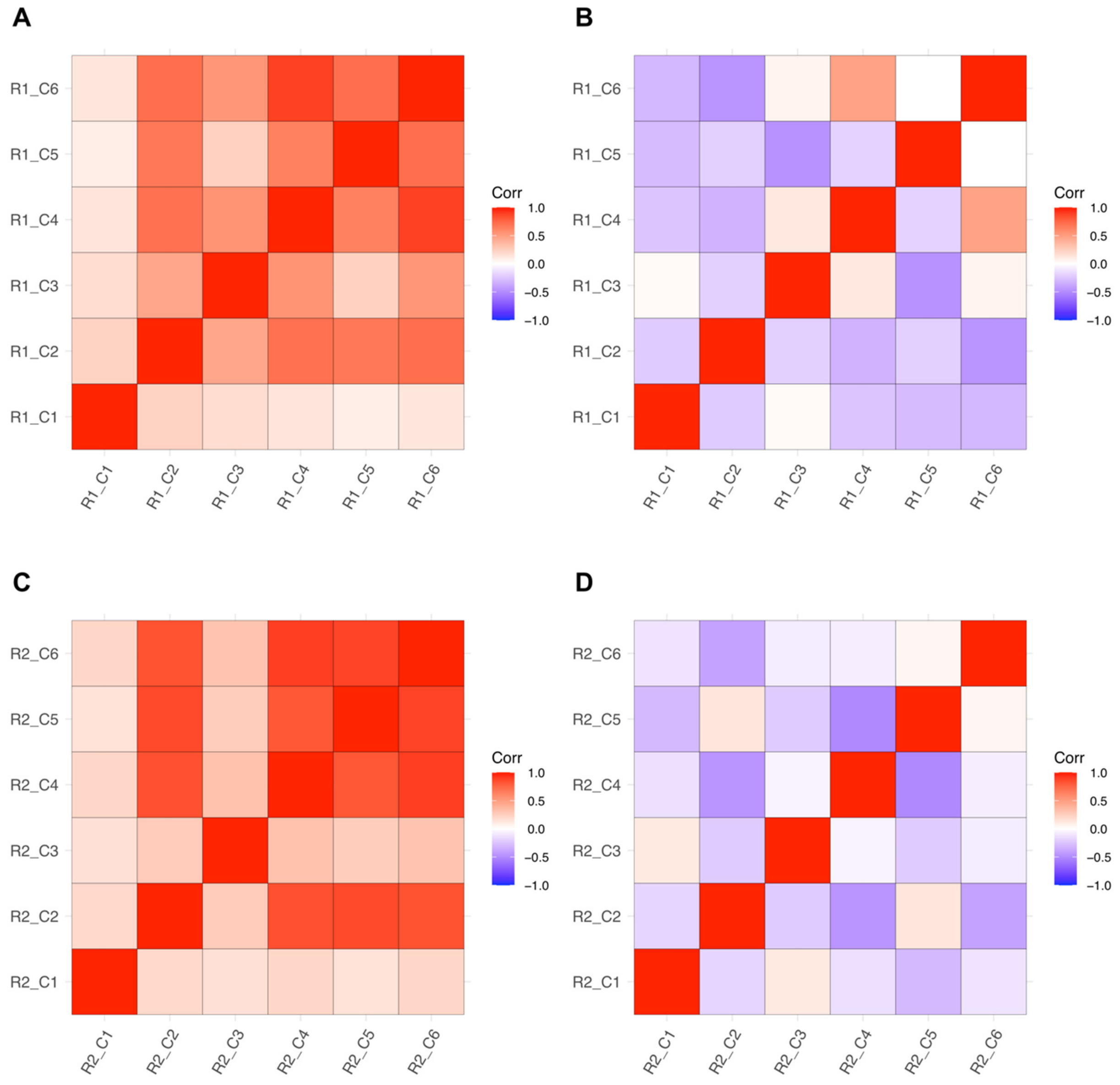

Figure S2. Correlation between RPE clusters. (**A**) Pearson correlation among R1 RPE cell clusters using the average expression of genes in each cluster. (**B**) Pearson correlation among R1 RPE cell clusters using differential expression (log_2FC) of genes in each cluster relative to cells in other clusters. (**C**) Pearson correlation among R2 RPE cell clusters using average expression of genes in each cluster. (**D**) Pearson correlation among R2 RPE cell clusters using differential expression of genes in each cluster relative to cells in other clusters. Positive correlations are shown in red and negative correlations in blue. Correlation with nominal p-value < 0.05 are considered significant.
