# Supplementary material for: Single-Cell RNA Sequencing Reveals Molecular Features of Heterogeneity in the Murine Retinal Pigment Epithelium
Source: Int J Mol Sci. 2022 Sep 8;23(18):10419. doi: 10.3390/ijms231810419 (PMC9499471; doi:10.3390/ijms231810419)
Supplement: Supplementary file 1 [file ijms-23-10419-s001.zip › Figure S3.pdf]

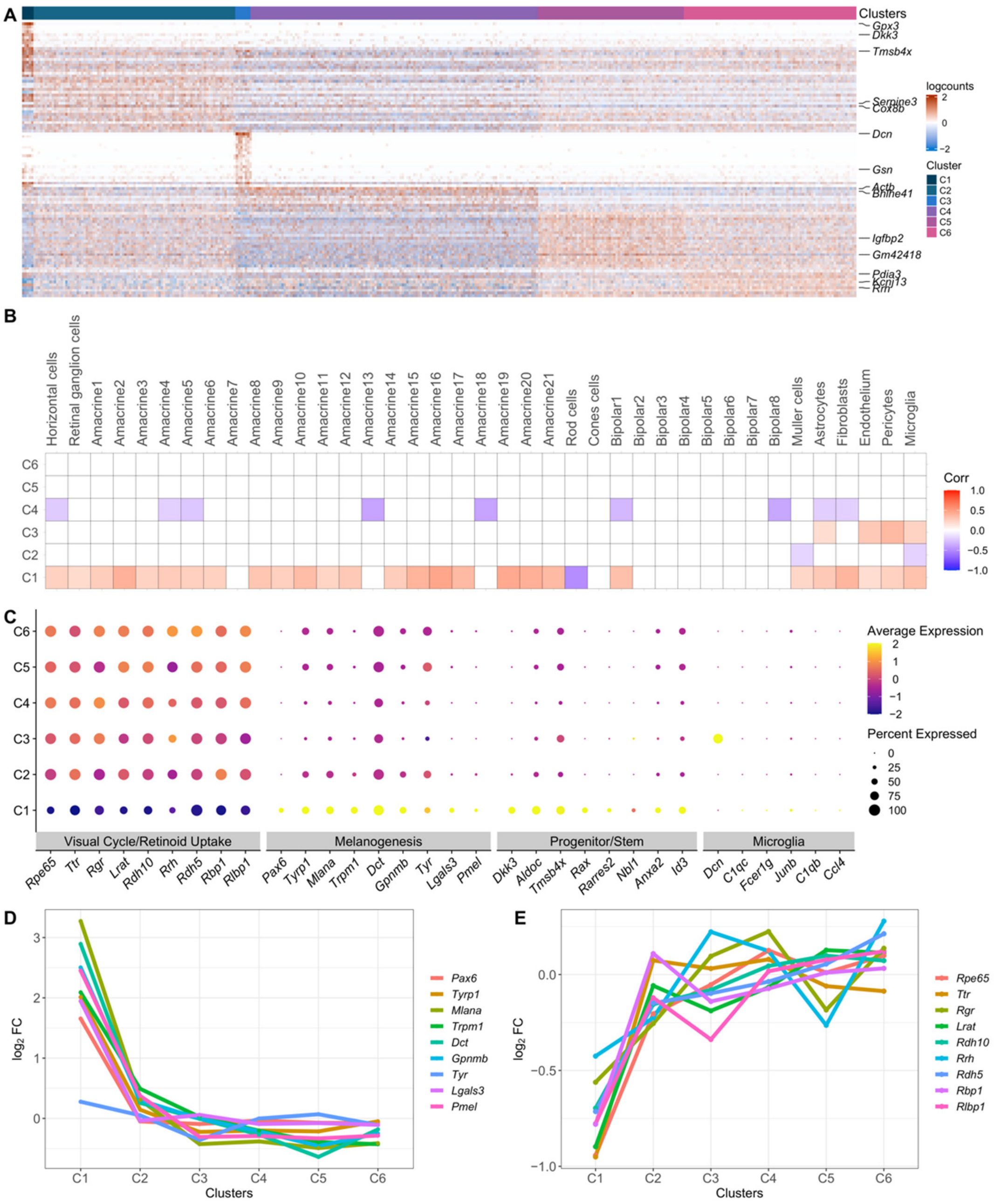

Figure S3. Heterogeneity of R2 RPE cell populations. (**A**) Top 20 differentially expressed genes in clusters, ranked by FDR, are shown in the heatmap. Gene expression values were centered, scaled, and transformed to a scale from −2 to 2. Select signature genes are highlighted on the right. (**B**) Correlation between single cell clusters from R2 and microglial retinal cell clusters**.** Pearson correlation coefficients were calculated log fold change in expression of genes in each cluster**.** Positive correlations are shown in red and negative correlations in blue. Correlation with nominal p-value < 0.05 are considered significant and shown in figure. (**C**) Dot plot showing marker gene expression for different RPE specific pathways (visual cycle, melanogenesis), and cell types (stem cell and immune cells). Dot sizes indicate the percentage of cells in each cluster expressing the gene, and colors indicate average expression levels. (**D**) Differential expression (log_2FC) of melanogenesis genes along RPE clusters C1–6. (**E**) Differential expression (log_2FC) of visual cycle genes in C1–6.
