# Supplementary material for: Single-Cell RNA Sequencing Reveals Molecular Features of Heterogeneity in the Murine Retinal Pigment Epithelium
Source: Int J Mol Sci. 2022 Sep 8;23(18):10419. doi: 10.3390/ijms231810419 (PMC9499471; doi:10.3390/ijms231810419)
Supplement: Supplementary file 1 [file ijms-23-10419-s001.zip › Figure S4.pdf]

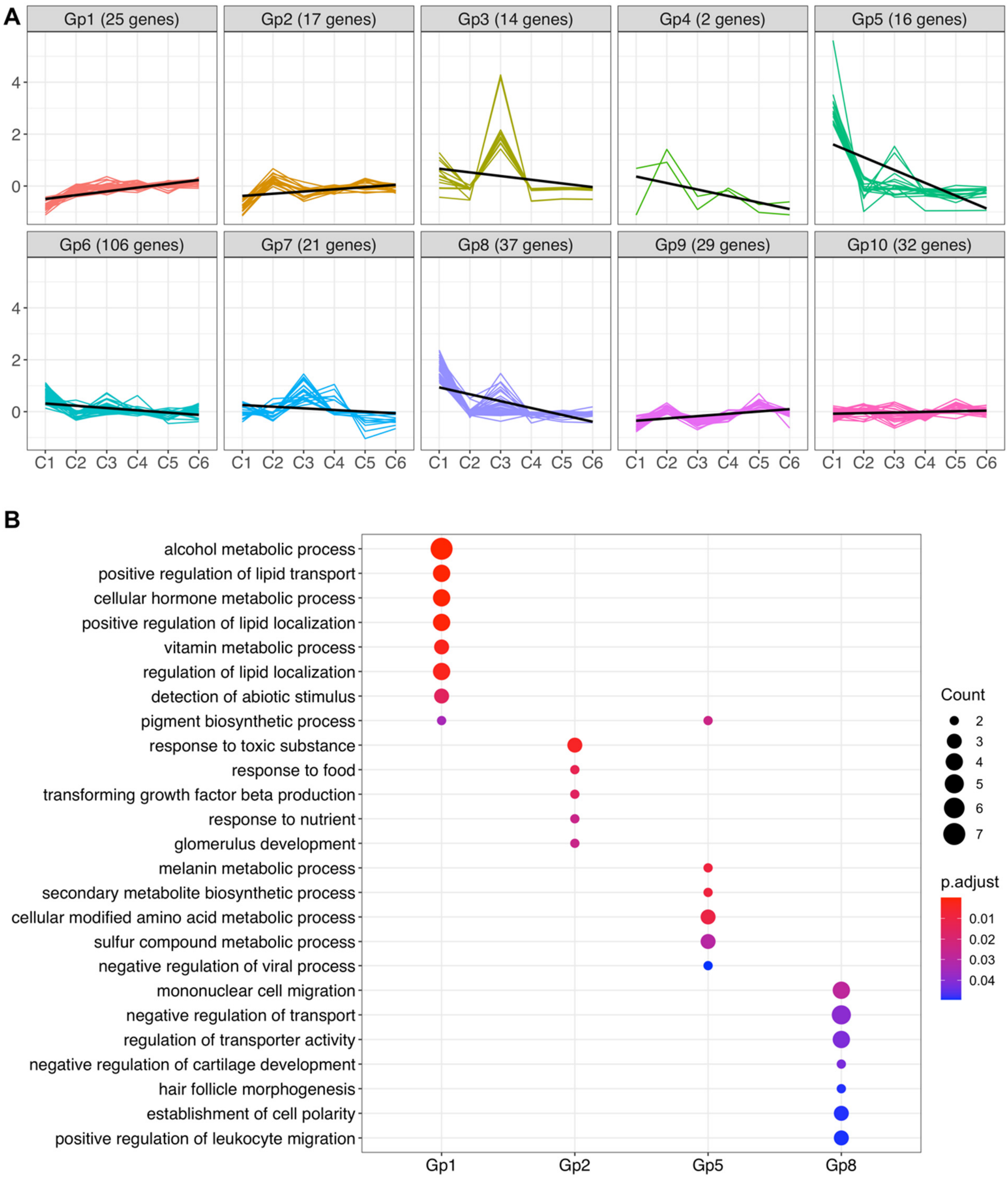

Figure S4. Characterization of R2 RPE subpopulations. (**A**) k-means clustering of differentially expressed genes in R2 RPE cell populations. Clustering analysis identified 10 group of genes with distinct expression profiles across possible maturation timeline, each plotted in a different color. The number of genes in each group are shown in parentheses. (**B**) Enrichment of biological processes in selected groups using clusterprofiler. The significance threshold for all enrichment analyses was set to 0.05 using Benjamini-Hochberg corrected p-values.
