# Supplementary material for: Single-Cell RNA Sequencing Reveals Molecular Features of Heterogeneity in the Murine Retinal Pigment Epithelium
Source: Int J Mol Sci. 2022 Sep 8;23(18):10419. doi: 10.3390/ijms231810419 (PMC9499471; doi:10.3390/ijms231810419)
Supplement: Supplementary file 1 [file ijms-23-10419-s001.zip › Table S1.pdf]

Human Native RPE Transcriptome Studies

Samples	Platform	Results	Reference
Normal ocular tissues from the periphery and posterior pole were obtained from a 44-year-old male and 88-year-old female undergoing exenteration surgery. Following the removal of retina fragments, RPE was harvested by gentle scraping and frozen within 45 min of surgery.	SAGE	Excluding singleton tags, 10,404 unique RPE transcripts assigned to 6,401 known genes were identified. Of 1,000 highly expressed RPE tags, 227 were of known function. Transcripts from protein degradation genes constituted roughly 10% of the total, consistent with the prominent phagocytic activity of the RPE.	[35]
Eyes from a 53-year-old male and a 12-year-old female donor were obtained within several h after death. The retina was removed from the posterior eyecup and RPE sheets were released by a 2 h incubation with dispase.	EST analysis	A nonredundant set of >1,100 RPE-expressed genes was identified from amplified and unamplified EST libraries.	[36]
Morphologically normal RPE cells (5,000) from 15 human globes from donors (ages 52–82) were isolated by laser capture (< 7 h postmortem). cDNAs were hybridized to a 4.3k microarray.	Microarray	RPE gene expression was similar in the macula and periphery, although 11 genes had lower expression in the macula. Roughly 26% of highly expressed genes were involved in protein synthesis and degradation.	[37]
RPE cells from one macular and two peripheral fragments were isolated by laser capture from cryosectioned eyes (< 24 h postmortem) of 6 healthy human donors (ages 17–36). cDNAs were hybridized to a 22k microarray.	Microarray	438 genes were differentially expressed between macular and peripheral regions. Validation of 33 genes using RT-PCR showed an 84% correlation in fold-change. 1–5% of the RPE transcriptome was differentially expressed depending on topographical location.	[38]
Macular RPE cells were isolated by laser capture from cryosectioned eyes (< 30 h postmortem) from 6 healthy human donors (ages 63–78). cDNAs were hybridized to a 22k microarray.	Microarray	19,746 transcripts were found to be significantly expressed in the macular RPE. Functional analysis of abundant transcripts revealed enrichment for oxidative phosphorylation, ATP synthesis, and phosphatidyl-inositol metabolism. Individual variation in the complement cascade was noted.	[39]
RPE, choroid, and photoreceptor layers were isolated by laser capture from 5 human eyes ages 63–78 (16–22 h postmortem). cDNAs were hybridized to a 44k microarray.	Microarray	114 RPE-specific genes were identified by excluding transcripts from retina and choroid. Of 39 selected for validation, 85% were validated through literature and PCR confirmed RPE expression for the remaining genes.	[40]
Native RPE and choroid was prepared from 4 adult (ages 64–89), 4 fetal donors (< 12 h postmortem) or cultured RPE samples and analyzed with a 55k microarray.	Microarray	A total of 154 RPE signature genes was identified. Using qRT-PCR, 48 genes were highly expressed both in vivo and in vitro. Cell culture was found to change expression but did not affect the enrichment of signature genes.	[41]
RPE cells were obtained from digestion of fetal ocular tissue and manual cell isolation.	scRNA-Seq	A total of 10,074 cells was obtained with 5,522 median genes per cell. Cell cluster analysis yielded two clusters assigned to macular and peripheral regions, Additional functional heterogeneity was identified in two macular and eight peripheral subpopulations.	[46]
RPE/choroid samples were dissected from the macula and periphery of three donor eyes (4–8 h postmortem). The tissue was incubated in papain and pipetted to obtain cells.	scRNA-Seq	Differential expression was noted between macular and peripheral RPE.	[47]

Human Native RPE Transcriptome Studies

Samples	Platform	Results	Reference
RPE was obtained from the posterior tissue of 3 donor eyes (12–24 h postmortem) by incubation in trypsin.	scRNA-Seq	A total of 10,074 cells was obtained with 5,522 median genes per cell. Cell cluster analysis yielded two clusters assigned to macular and peripheral regions, Additional functional heterogeneity was identified in two macular and eight peripheral subpopulations.	[48]

Mouse Native RPE Transcriptome Studies

Samples	Platform	Results	Reference
Ocular tissue from C57BL/6JOlaHsd mice at 5 months of age (n = 3) was dissected by laser microdissection to yield RPE, photoreceptor cells and choroid. Transcriptomic datasets were compared with a previous studies of human donor eyes [39-41]. Signature genes were identified with correction for possible RNA contamination from RPE-adjacent layers.	Microarray	RPE signature genes were identified in mouse (64), human (171), and shared between the two species (22). In general, the mouse and human RPE were found to be very similar, although species-specific functional differences were noted, including in pathobiological pathways.	[42]
Mice were examined at six time points during the light-dark cycle (Zeitgeber time 0, 2, 4, 9, 14, and 19 h). RPE from n = 5 male C57BL/6NCrl mice (10–13 weeks of age) at each time point was scraped from peeled eyecups in RNA*later*.	RNAseq	Across all time points, < 75% of the transcripts originated from the RPE, while > 22% originated in the choroid. 756 significant differentially expressed genes were identified that varied with the light-dark cycle. Energy metabolism genes in the RPE fall under circadian control. The highest energy demand occurred at night and was met by using glycogen and glucose but not fatty acids as energy sources.	[43]
Mice were examined at six time points during the light-dark cycle (Zeitgeber time 0, 2, 4, 9, 14, and 19 h). RPE in n = 5 male C57BL/6NCrl mice (10–13 weeks of age) per time point was scraped from peeled eyecups in RNA*later*.	RNAseq	Cell purity of the preparation was the same as in a related article [43]. Expression of genes encoding phagocytosis, intracellular trafficking, actin cytoskeleton, and tight junction proteins were found to vary with circadian rhythm.	[44]
The RPE from C57BL/6J (n = 3) mice reared in a light-dark cycle and then kept in the dark for 3 d was harvested at two time points to assess the circadian control of phagocytosis. Eyecups were prepared in dim red light, retinas were peeled, RNA*later* was added, and brief sonication was used to release the RPE.	RNAseq	14,083 transcripts were shared at the two time points; 20% of protein-coding transcripts varied. Post-hoc filtering indicated that < 2% of photoreceptor- specific genes and < 0.1% of choroid-specific genes were present. Phagocytic pathways such as integrin signaling, cytoskeleton signaling, and adhesion junction signaling were under circadian control. Mitochondrial respiration was upregulated during phagocytosis and was involved in circadian control.	[45]
